# Removing the sporoderm from the sporoderm-broken spores of *Ganoderma lucidum* improves the anticancer and immune-regulatory activity of *the* water-soluble polysaccharide

**DOI:** 10.3389/fnut.2022.1006127

**Published:** 2022-09-16

**Authors:** Liu Fang, Qian Zhao, Cuiling Guo, Dandan Guo, Zhenhao Li, Jing Xu, Chengjie Guo, Tingting Sang, Ying Wang, Jiajun Chen, Chaojie Chen, Rong Chen, Jianjun Wu, Xingya Wang

**Affiliations:** ^1^School of Pharmaceutical Science, Zhejiang Chinese Medical University, Hangzhou, China; ^2^Zhejiang Engineering Research Center of Rare Medicinal Plants, Wuyi, China

**Keywords:** *Ganoderma lucidum*, polysaccharide, cancer, splenomegaly, inflammation, macrophage

## Abstract

Plant-derived polysaccharides have demonstrated promising anti-cancer effects *via* immune-regulatory activity. The aim of the current study was to compare the chemical property and the anticancer effects of polysaccharides extracted from the sporoderm-removed spores of the medicinal mushroom *Ganoderma lucidum* (RSGLP), which removed the sporoderm completely, with polysaccharides extracted from the sporoderm-broken spores of *G. lucidum* (BSGLP). We found that RSGLP has a higher extraction yield than BSGLP. HPGPC and GC-MS results revealed that both RSGLP and BSGLP are heteropolysaccharides, but RSGLP had a higher molecular weight and a different ratio of monosaccharide composition than BSGLP. MTT and flow cytometry results demonstrated that RSGLP exhibited much higher dose-efficacy in inhibiting cell viability and inducing apoptosis than BSGLP in 8 cancer cell lines representing colon (HCT116 and HT29), liver (HepG2 and Huh-7), breast (MDA-MB-231 and MCF-7), and lung cancers (NCI-H460 and A549). Furthermore, RSGLP is more effective in inhibiting HCT116 and NCI-H460 xenograft tumor growth and inhibiting tumor-induced splenomegaly than BSGLP in nude mice, suggesting a better effect on regulating immunity of RSGLP. Next, we found that RSGLP is more potent in inhibiting the level of serum inflammatory cytokines in nude mice, and in inhibiting the activation of macrophage RAW264.7 and the expression of the inflammatory mediators IL-1β, TNF-α, iNOS, and COX-2 *in vitro*. This is the first study to compare the chemical properties, anti-cancer, and immune-regulatory effects of RSGLP and BSGLP using multiple cancer cell lines. Our results revealed that the sporoderm-removed spores of *G. lucidum* (RSGL) and RSGLP may serve as new anticancer agents for their promising immune-regulatory activity.

## Introduction

Cancer is a leading cause of death worldwide. Among the common cancers, lung, breast, colon, and liver cancers all rank in the top six for both incidence and mortality globally (1). At present, the causal relationship between inflammation, innate immunity, and carcinogenesis has been widely recognized (2). It is generally believed that inflammatory immune responses play important roles at different stages of tumor development and progression (2). In recent years, natural products are becoming increasingly important in the prevention or therapy against cancers in particular, for their anti-inflammatory and immunoregulatory effects (3, 4).

*Ganoderma lucidum* (*G. lucidum*) is a widely and globally distributed medicinal mushroom, which has been broadly studied by agricultural, food, and pharmacological industries (5). *G. lucidum* has been recognized to have many biological activities including antidiabetic, immunoregulatory, and anticancer effects (6–8). Studies have shown that *G. lucidum* contains more than 400 kinds of bioactive substances, including polysaccharide, triterpenoids, sterols, amino acids, etc (9). Among these compounds, *G. lucidum* polysaccharide (GLP) has been extensively studied, which has a variety of pharmacological effects, such as anti-tumor, anti-oxidation, anti-inflammatory, liver protection, and immunoregulatory activities as reviewed before (10–13). Many studies have demonstrated that GLP elicits anticancer effects in several cancers (14–19).

The chemical structure and composition of polysaccharides are diverse. The differences in chemical properties have certain influences on its pharmacological action, such as molecular weight (Mw), monosaccharide composition, α/β configuration, glycosidic bond, the side chain length, and branching degrees (20, 21). Other than the above factors, the anticancer effects of GLP were also affected by the source of *G. lucidum*, extraction methods, and type of GLP, eg. extracted from which life cycle of *G. lucidum*. At present, most studies examining the biological activities of *G. lucidum* or GLP have been focused on the fruiting bodies or the mycelium of *G. lucidum*. Another important part of the life cycle of *G. lucidum* is the spores. The sporoderm (spore wall) of *G. lucidum* spores is composed mainly of chitin, calcium, and silicon, which is very difficult to be broken down (22). The sporoderm breaking technology resulted in the release of the bioactive components in the spores of *G. lucidum*, which has been widely utilized and has been reported to improve both the amount of the bioactive contents and the efficacy of the biological activities of the spores (23, 24). Our previous studies reported that GLP extracted from BSGL significantly inhibited proliferation, induced apoptosis, and inhibited autophagic flux in prostate or colorectal cancer cells (18, 25, 26).

However, BSGL still contains a large number of the hard outer bilayer of the sporoderm (22, 27). With the development of extraction and purification technology, more advanced technologies have been applied to obtain high-quality G. lucidum products. Lin et al. have mentioned in “Lingzhi and Health” (28) that a recent Chinese patent (CN201310743712.8) developed a combined method of breaking the spores by supersonic air jet milling, solvent extraction, and filtration to remove the sporoderm completely. This new technology yielded the high-quality spore product of G. lucidum, so-called sporoderm-removed spores of G. lucidum (RSGL). Recently, the composition of triterpenoids in BSGL and RSGL was compared for the first time that RSGL contained much higher contents of triterpenoids than BSGL (27). In addition, RSGL demonstrates better immunoregulatory activities than BSGL in Vinorelbine-induced immune-deficient Zebrafish model (27). We recently reported that RSGLP induced apoptosis in human gastric cancer AGS cells *via* disruption of autophagic flux. However, at present, the content, Mw, and other chemical properties of polysaccharides in RSGL have never been characterized and the biological function of RSGLP against cancer is still largely unknown.

Therefore, the aim of the current study was to first compare the extraction yield and chemical properties of crude RSGLP and BSGLP. Next, we aimed to compare the efficacy of the cytotoxicity and pro-apoptotic activities of RSGLP and BSGLP in 8 cancer cell lines representing colon, liver, breast, and lung cancers. We also compared the anti-tumor, immune-regulatory, and anti-inflammatory effects of RSGLP and BSGLP using xenograft mouse models and macrophage RAW264.7 cellular model. To our knowledge, this is the first study to characterize the chemical property of RSGLP and to study the anti-cancer and anti-inflammatory effects of RSGLP.

## Materials and methods

### Materials

The fluorescein isothiocyanate (FITC)-Annexin V and propidium iodide (PI) apoptosis kit was obtained from BD Pharmingen (San Diego, CA, USA). MTT was obtained from AMRESCO (Solon, OH, USA). Lipopolysaccharides (LPS) was purchased from Sigma (St. Louis, MO, USA). Mouse interleukin 6 (IL-6) (#MM-0163M1), tumor necrosis factor (TNF-α) (#MM-0132M1), and interleukin 1β (IL-1β) (#MM-0040M1) enzyme-linked immunosorbent assay (ELISA) kits were obtained from Jiangsu Meimian Industrial Co. Ltd (Jiangsu, China). The RNA extraction kit was obtained from Aidlab Biotechnologies, Co. Ltd. (Beijing, China). The iScript cDNA synthesis kit and SYBR Green master mix were obtained from Bio-Rad Laboratories, Inc. (Hercules, CA, USA). The polyclonal TNF-α (#db4312) antibody was purchased from Diagbio Technology (Hangzhou, China). The polyclonal IL-1β (#bs-6319R) antibody was purchased from Bioss Technology (Beijing, China). The polyclonal β-actin (#4967) and the anti-rabbit secondary antibodies were purchased from Cell Signaling Technology (Danvers, MA, USA). The iNOS (#AF0199) and COX-2 (#AF7003) antibodies for immunofluorescence and Western blotting were obtained from Affinity Biosciences (Jiangsu, China). The goat anti-rabbit secondary antibody (#ab150077) for immunofluorescence staining was obtained from Abcam (Cambridge, UK). The PCNA antibody (#GB13010) and the anti-goat IgG secondary antibody (#GB23303) for immunohistochemistry (IHC) were obtained from Wuhan Goodbio Technology Co., Ltd. (Wuhan, China). Terminal deoxynucleotidyl transferase (TdT)-mediated dUTP nick end-labeling (TUNEL) assay kit was obtained from Roche Applied Sciences (Basel, Switzerland).

### Extraction, purification, and molecular weight determination

The powder of RSGL and BSGL was obtained from Shouxiangu Institute (Wuyi, Zhejiang, China). Water extraction and alcohol precipitation were used to extract the polysaccharides as described before (29). After hot water extraction, six volumes (v/v) of cold 95% ethanol were added to precipitate polysaccharide for 24 h. The sediment was separated and dissolved in a certain amount of ultrapure water and the crude polysaccharides were de-proteinized through the Sevag method and dialyzed using 3 kDa molecular weight cut-off membrane for the removal of unwanted small molecules. The water-soluble polysaccharides were then freeze-dried in an H051 freeze dryer (LaboGene, Lynge, Denmark). High-performance liquid gel chromatography (HPGPC) using an Agilent 1,100 HPLC system (Agilent, Santa Clara, CA, USA) combined with KS-804 and KS-802 columns (8.0 mm × 300 mm; Shodex Co., Tokyo, Japan) was employed to evaluate the purity and Mw of the two GLPs. The specific condition was dissolving 2 mg of sample powder into a 2 mg/mL solution by using a 0.2 M NaCl aqueous solution as mobile phase, followed by centrifugation, then the supernatant was taken and injected into the columns at 20 μL. Finally, calculate the Mw based on dextran curve, and the preliminary calibration of column was conducted using different pullulans of known molecular weight (P-5, P-10, P-20, P-50, P-100, P-200, P-400, and P800, Shodex Co.).

### Sugar and protein contents, and monosaccharide composition determination

The total sugar content was determined by the phenol–sulphuric acid colorimetric method and glucose was used as reference standard for polysaccharide determination. Protein content was determined using Pierce BCA protein quantification kit with bovine serum albumin (BSA) as standard according to manufacturer's instruction. Monosaccharide composition was analyzed by Agilent 7890B/5977A gas chromatography-mass spectrometry (GC-MS) analysis using the TR-5MS chromatography column (60 m × 0.25 mm × 0.25 μm) from Thermo Fisher Scientific, Inc. (Grand Island, NY, USA). Briefly, a total of 2 mg of samples were hydrolyzed with 2 M trifluoroacetic acid (TFA) (3 mL) at 121°C for 2 h, and methanol was used to remove TFA completely. NaBH4 and water were added to reduce the residue for 5–8 h. After the samples were dried at 105°C for 10 min, it was acetylated by acetic anhydride at 105°C for 1 h. Finally, the monosaccharide compositions of RSGLP and BSGLP were measured by GC-MS method as described before (29).

### Fourier transform infrared spectroscopy analysis

FT-IR was carried out to identify the functional groups in GLP. Briefly, 2 mg of GLP was mixed with 200 mg of KBr into a 1 mm pellet. The FT-IR spectrum of GLP was recorded in the wave range of 400–4,000 cm-1 on a PerkinElmer 2,000 FT-IR spectrometer (Waltham, MA, USA).

### Cell culture

Human colorectal cancer cell line HCT116 and HT-29, and the macrophage cell line RAW264.7 were purchased from the American Type Culture Collection (ATCC, Manassas, VA, USA). Human liver (HepG2), breast (MDA-MB-231 and MCF-7), lung (A549 and NCI-H460), and the liver HuH-7 cancer cell lines were purchased from Fenghui Biotechnologies Inc. (Changsha, China). All cells were maintained in a humidified incubator containing 5% CO_2_ at 37°C. HCT116, HT-29, HepG2, HuH-7, MDA-MB-231, MCF-7 and RAW264.7 cells were cultured in DMEM, while A549 and NCI-H460 cells were cultured in RPMI 1,640, supplemented with 10% fetal bovine serum (FBS) and 1% penicillin-streptomycin.

### Cell viability assay

Cell viability was measured using MTT assay. Briefly, HCT116, HT-29, HepG2, HuH-7, MDA-MB-231, MCF-7, A549, and NCI-H460 cells were seeded in 96-well plates at a density of 1 × 10^4^ cells/well and cultured in a 37°C incubator. A pilot study suggested that it required lower concentrations of RSGLP than BSGLP to kill cancer cells. Therefore, we used 0, 1.25, 1.875, 2.5, or 3.125 mg/mL of RSGLP, and used 0, 5, 7.5, 10, or 12.5 mg/mL of BSGLP based on results from the pilot study. When cells reached approximately 50% confluence, medium was removed and cells were treated with RSGLP (0–3.125 mg/mL) or BSGLP (0–12.5 mg/mL) for 24, 48 or 72 h. MTT assay was conducted as described before (26).

### Flow cytometric analysis of cell apoptosis

Cell apoptosis was determined by flow cytometric assay as described before (26). Briefly, cells were seeded at a density of 2 × 10^5^ cells/well in 6-well plates and incubated in a 37°C incubator. When cells were about 50% confluence, they were treated with 0–3.125 mg/mL RSGLP or 0–12.5 mg/mL BSGLP for 48 h. Then the cells were digested and washed twice with PBS. PI and Annexin V were added to the cell solution and stained in the dark for 15 min. The samples were measured by the Merk Guava EasyCyte flow cytometer system (Darmstadt, Germany) and the percentage of the total apoptosis cells was analyzed by adding the rate of Annexin V+/PI- (early apoptosis) positive and rate of Annexin V+/PI+ (late apoptosis) positive cells.

### *In vivo* tumor xenograft study

All the experimental procedures were conducted following the Guide for the Use and Care of Laboratory Animals of the National Institutes of Health. This study was approved by the Committee on the Ethics of Animal Experiments of Zhejiang Chinese Medical University (Permit Number: SYXK 2018-0012). Four-week-old Male BALB/c nude mice (5 wk old) were housed in specific pathogen-free (SPF) environment and were randomly divided into control, model (MOD) and GLP treatment groups: low-dose of RSGLP or BSGLP group (150 mg/kg), high-dose of RSGLP or BSGLP group (300 mg/kg) by body weight (*n* = 8 per group). NCI-H460 cells (5 × 10^6^ cells in 200 μL PBS) and HCT116 cells (5 × 10^6^ cells in 200 μL PBS) were injected subcutaneously into the right flank of each mouse. From the day after the injection, the mice were gavaged every day with both GLPs. Mice were weighed every day, while tumor volumes were measured twice a week using a digital vernier caliper (0.01 mm). The formula used to calculate tumor volume was: V (mm3) = length × width^2^/2. At the end of the study, all mice were sacrificed by CO_2_. Blood was collected *via* cardiac puncture method, and the serum was collected by centrifugation at 13,000 rpm at 4°C.

### Immunohistochemistry and TUNEL staining

Tumor tissues (5 μm) were deparaffinized before using citric acid buffer (pH 6.0) to retrieve the antigens. Next, the sections were incubated with 3% H_2_O_2_ for 5–10 min to eliminate endogenous peroxidase activity, then blocked in 1% BSA for 25 min before incubating overnight with PCNA (1:2000) primary antibody at 4°C. Next, the slides were incubated in biotinylated anti-goat IgG secondary antibody (1:200) for 50 min at room temperature. Next, DAB was added for color development, and hematoxylin was re-dyed for 3 min, then dehydrated, sealed, and images were taken. For TUNEL staining, the tumor tissues were stained using the TUNEL kit according to the manufacturer's instructions.

### ELISA

The levels of inflammatory cytokines (IL-6, TNF-α, and IL-1β) in the serum were measured by ELISA kits according to the manufacturer's instructions. The OD value of each well was detected at 450 nm using multi-well plate reader from Bio-Tek Instruments Inc., and the content of each cytokine in the serum was calculated according to the standard curve.

### Macrophage LPS stimulation assay

LPS (1 μg/mL) was used to activate macrophage RAW264.7 cells. In addition, cells were treated with different concentrations (0, 0.625, 1.25, and 2.5 mg/mL, concentrations without causing cytotoxicity) of RSGLP or BSGLP for 24 h together with 1 μg/mL LPS.

### Immunofluorescence staining

RAW264.7 cells were seeded in 6-well plates at a density of 2 × 10^5^ cells/well. At 50% confluence, cells were treated with LPS with or without 1.25 mg/mL RSGLP or BSGLP for 24 h. At the endpoint, cells were fixed in 4% paraformaldehyde solution and 0.5% Triton solution was added to penetrate the cytomembrane. After blocking with 2% BSA, cells were incubated overnight at 4°C with iNOS or COX-2 antibodies (1:500), followed by incubation with fluorescent secondary antibody (1:1000) at 37°C for 1 h. The nucleus was stained with DAPI. All images were taken using a laser scanning confocal microscope (LSM880, Carl Zeiss, Germany).

### RNA isolation and quantitative real-time PCR

Total RNA from RAW264.7 cells were prepared according to the manufacturer's instruction. A total of 1 μg RNA was reverse transcribed to cDNA using iScript Reverse transcription supermix. qRT-PCR reactions were performed using SYBR Green master mix on CFX96 Real-Time PCR system. β-Actin was used as the reference. The fold change of relative expression was calculated by 2-ΔΔCt method. Primer sequences are shown in Supplementary Table 1.

### Western blotting

Total protein was extracted from cells using the standard methods. An equal amount of total protein (25 μg) was resolved by SDS-PAGE gel and transferred to PVDF membranes. After blocking with 5% nonfat milk in TBST (Tris-buffered saline) for 1 h at room temperature, the membranes were incubated overnight with the primary antibodies (1:1000) at 4°C. Next day, the membranes were incubated with anti-rabbit secondary antibody (CST, 1:2000) for 1 h. The signals were captured using ECL chemiluminescence reagent from PerkinElmer (Waltham, MA, USA) and detected using Minichemi TM610 chemical imaging System (Beijing, China). The optical density was quantified using ImageJ software Version 1.41 (Bethesda, Maryland, USA).

### Statistical analysis

All data were stated as the mean ± standard error (SE) from at least three independent experiments or samples. Statistical analyses were performed using a one-way or two-way ANOVA for multiple comparisons followed by Dunnett's test using SPSS software 17.0 (Chicago, IL, USA). A *p*-value of < 0.05 was considered to be statistically significant. The figures were plotted using GraphPad Prism 7.04 software (La Jolla, CA, USA).

## Results

### Extraction yields and chemical properties of RSGLP and BSGLP

**Figure 1:**
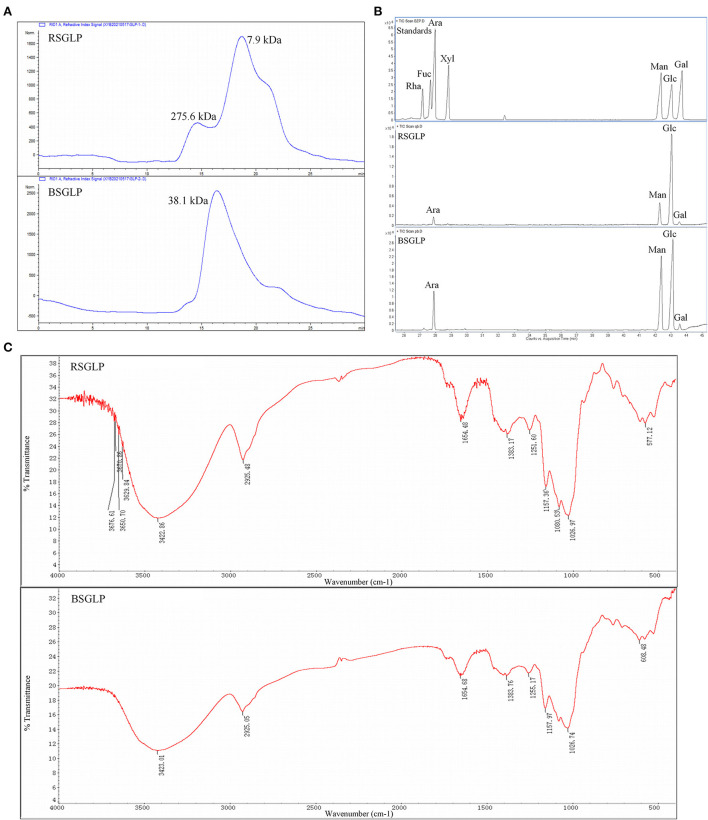
Chemical characterization of RSGLP and BSGLP. **(A)** The HPGPC chromatogram of standards, RSGLP, and BSGLP to determine molecular weight. **(B)** The GC-MS analysis of the monosaccharides composition in RSGLP and BSGLP. The standards Rha, Fuc, Ara, Xyl, Man, Glu, and Gal were located in the order of retention time as shown in the GC–MS chart. **(C)** FT-IR spectrum of RSGLP and BSGLP.

The results showed that the extraction yield of the crude water-soluble RSGLP (23.7%) is about 8.78 times that of the crude water-soluble BSGLP (2.7%) under the same conditions. The total carbohydrate contents of RSGLP and BSGLP was 83.3 and 67.5% as determined by the phenol-sulfuric acid colorimetric method. Protein content was determined to be 2.1 and 4.2% in RSGLP and BSGLP using BCA assay. The average Mw distributions of RSGLP and BSGLP were determined as shown in Figure 1A. RSGLP showed two peaks with Mw of 275.6 kDa (22.01%) and 7.9 kDa (77.99%). BSGLP showed one significant peak with Mw of 38.1 kDa (100%) (Figure 1A). The results suggest that RSGLP contained more polysaccharides with larger Mw than BSGLP. And the average Mw of RSGLP and BSGLP were calculated to be 66.8 kDa and 38.1 kDa, respectively.

According to the retention time of alditol acetate derivatives of standards, both RSGLP and BSGLP were heteropolysaccharides containing the same four monosaccharides: arabinose, mannose, glucose, and galactose, but different in component proportion (Figure 1B). RSGLP consisted of arabinose, mannose, glucose, and galactose in the relative content of 4.19: 15.69: 78.15: 1.97. BSGLP consisted of arabinose, mannose, glucose, and galactose in the relative content of 11.65: 35.54: 50.58: 2.23. Like RSGLP, BSGLP also contained highest amount of glucose, suggesting that glucose was the predominant structural monosaccharide of RSGLP and BSGLP. However, the ratio of glucose in BSGLP was less than RSGLP and the ratios of mannose and arabinose in BSGLP were more than doubled compared to RSGLP.

As shown in Figure 1C, the typical absorption bands for polysaccharides centered at 3,423 cm−1 (O-H stretching vibration), 2,925 cm−1 (C-H stretching vibration) and 1,383 cm−1 (C-H bending vibration) were found in the FT-IR spectra of both of the GLPs. The absorption band centered at 1,654 cm-1 indicated the presence of protein. The absence of absorption signal at 1,740 cm−1 indicated that there was no uronic acid in the two GLPs.

### Comparison of the cytotoxic activities of RSGLP and BSGLP

**Figure 2:**
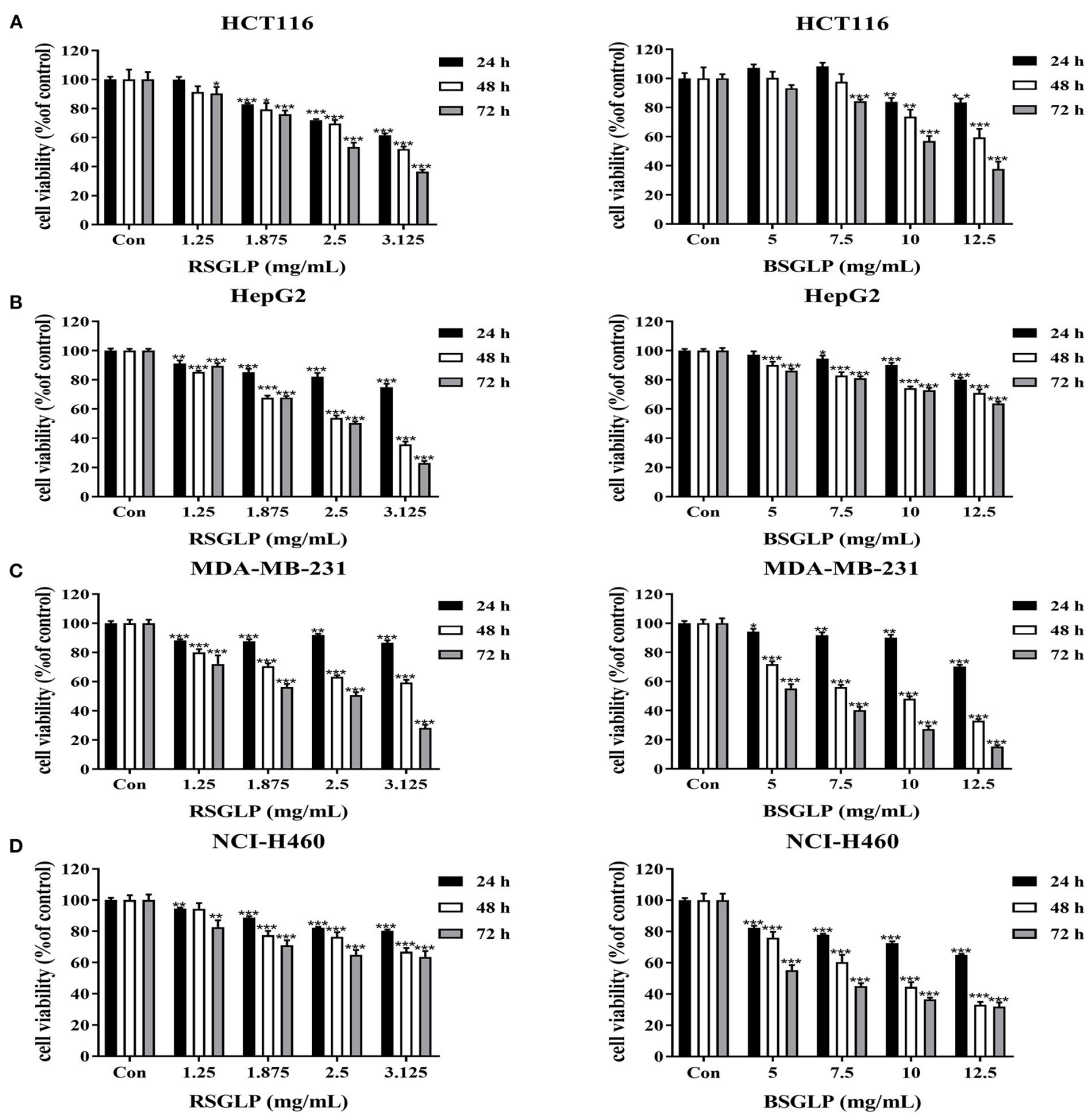
Comparison of the cytotoxic effects of RSGLP and BSGLP in colon, liver, breast, and lung cancer cells as determined using MTT assay. **(A)** HCT116. **(B)** HepG2. **(C)** MDA-MB-231. **(D)** NCI-H460. Cells were treated with different concentrations of RSGLP (0, 1.25, 1.875, 2.5, and 3.125 mg/mL) and BSGLP (0, 5, 7.5, 10, and 12.5 mg/mL) for 24, 48, and 72 h. Data are presented as mean ± SE from three independent experiments. *p < 0.05, **p < 0.01, ***p < 0.001, compared with control group.

We then compared the effects of RSGLP and BSGLP on cell viability using MTT assay in eight cancer cell lines, which represent colon, liver, breast, and lung cancers. MTT assay revealed that both RSGLP (0–3.125 mg/mL) and BSGLP (0–12.5 mg/mL) significantly inhibited the cell viability in colon HCT116, liver HepG2, breast MDA-MB-231, and lung NCI-H460 cancer cells upon increasing doses and time (Figures 2A–D). However, higher concentrations of BSGLP (5–12.5 mg/mL) were required to inhibit cell viability in these cells than RSGLP (1.25–3.125 mg/mL) (Figures 2A–D).

Compared to these four cancer cell lines, the other four cancer cell lines (colon HT-29, liver HuH-7, breast MCF-7, and lung A549) were more resistant to the two GLP treatments, as shown in the Supplementary Figures 1A–D. For example, at 24 h, both GLPs demonstrated no cytotoxic effects on all these four cell lines. RSGLP treatment only inhibited the cell viability of HT-29, HuH-7, and MCF-7 cells at 48 and 72 h (Supplementary Figures 1A–C). BSGLP treatments only inhibited the cell viability of HT-29 at 48 and 72 h (Supplementary Figure 1A), while the cell viability of HuH-7, MCF-7, and A549 were only inhibited at 72 h by the high concentrations of BSGLP (Supplementary Figures 1B–D). These results suggest that both GLPs could cause cytotoxicity in cancer cells, while RSGLP is more potent than BSGLP in inducing cell death. In addition, within the same cancer type, different cell lines respond differently to GLP treatments.

To compare the dose-efficacy of the two GLPs, IC_50_ was calculated by the GraphPad Prism 5. As shown in Table 1, at 24, 48, and 72 h, the IC_50_ of RSGLP was much lower than that of BSGLP in HCT116, HepG2, MDA-MB-231, and NCI-H460 cells. Other than these four cell lines, as shown in Supplementary Table 2, the IC_50_ of RSGLP and BSGLP were all increased in HT-29, HuH-7, MCF-7, and A549 cells. For certain cell line or time points, the IC_50_ values could not be calculated under current experimental conditions, which indicate that it requires much higher concentrations to induce cytotoxic effect in these cells (Supplementary Table 1). Taken together, these results demonstrate that RSGLP has a higher efficacy to inhibit cell viability than BSGLP, which is most likely affected by the differences of both the total polysaccharide content and the chemical properties of the polysaccharides in RSGL and BSGL, such as Mw and monosaccharide composition.

**Table 1:** Half-maximal inhibitory concentration (IC_50_) of RSGLP and BSGLP against the human cancer cell lines (HCT116, HepG2, MDA-MB-231, and NCI-H460).

		**IC**_**50**_ **(mg/mL)**			
	**HCT116**	**HepG2**	**MDA-MB-231**	**NCI-H460**
	24 h	3.554 ± 0.53	5.796 ± 1.22	-	6.464 ± 1.66
RSGLP	48 h	3.258 ± 0.38	2.557 ± 0.85	3.597 ± 0.92	4.336 ± 1.34
	72 h	2.629 ± 0.43	2.378 ± 0.73	2.264 ± 0.78	3.939 ± 1.28
	24 h	17.54 ± 1.27	19.49 ± 1.17	15.43 ± 1.24	19.47 ± 3.28
BSGLP	48 h	13.43 ± 1.54	22.55 ± 1.15	8.95 ± 1.54	9.089 ± 2.19
	72 h	10.94 ± 1.29	17.3 ± 1.23	6.48 ± 1.25	7.133 ± 2.34
	24 h	4.94	3.36	-	3.01
fold^a^	48 h	4.12	8.82	2.49	2.1
	72 h	4.16	7.28	2.86	1.81

All experiments were performed in triplicates and reported as mean ± SE. “−” The IC_50_ could not be calculated under the current treatment condition. “a” The IC_50_ of BSLGP divided by the IC_50_ of RSGLP at the same time point for each cell line.

### Comparison of the pro-apoptotic effects of RSGLP and BSGLP

**Figure 3:**
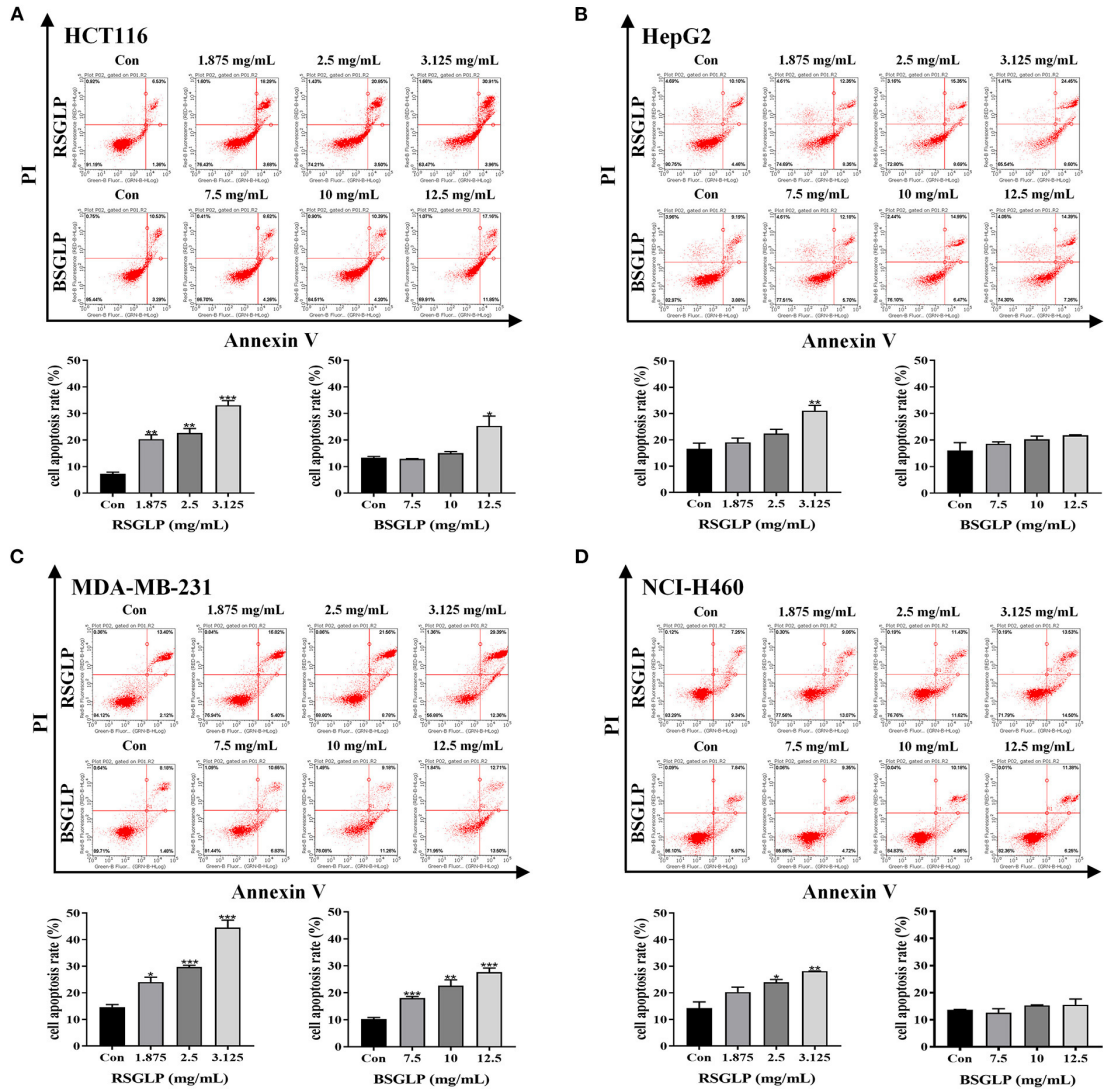
Comparison of the pro-apoptotic effects of RSGLP and BSGLP in colon, liver, breast, and lung cancer cells as determined using flow cytometry. **(A)** HCT116. **(B)** HepG2. **(C)** MDA-MB-231. **(D)** NCI-H460. Cells were treated with different concentrations of RSGLP (0, 1.875, 2.5, and 3.125 mg/mL) and BSGLP (0, 7.5, 10, and 12.5 mg/mL) for 48 h. The bottom panel is the total percentages of cell apoptosis. Data are presented as mean ± SE from three independent experiments. *p < 0.05, **p < 0.01, ***p < 0.001, compared with control group.

Flow cytometry analysis was performed to compare the effects of RSGLP (0–3.125 mg/mL) and BSGLP (0–12.5 mg/mL) in inducing apoptosis in the eight cancer cell lines. Consistent with cell viability results, treatment with RSGLP for 48 h induced apoptosis in all eight cancer cell lines, which showed stronger pro-apoptotic effects in HCT116, HepG2, MDA-MB-231, and NCI-H460 cells (Figures 3A–D), but weaker effects in HT-29, HuH-7, MCF-7, and A549 cells as shown in the (Supplementary Figures 2A–D). However, BSGLP induced apoptosis only in HCT116 and MDA-MB-231 cells and had no significant effect on the remaining cells at the current dose range (0–12.5 mg/mL) at 48 h (Figures 3A–D; Supplementary Figures 2A–D). These results indicate that after 48 h of treatment, RSGLP can induce apoptosis in eight cancer cell lines, while BSGLP can only induce apoptosis in HCT116 and MDA-MB-231 cells. Flow cytometry results further demonstrate that colon HCT116, liver HepG2, lung NCI-H460 and breast MDA-MB-231 cancer cells are more sensitive to GLPs than the other four cancer cells lines.

### Comparison of the antitumor effects of RSGLP and BSGLP *in vivo*

**Figure 4:**
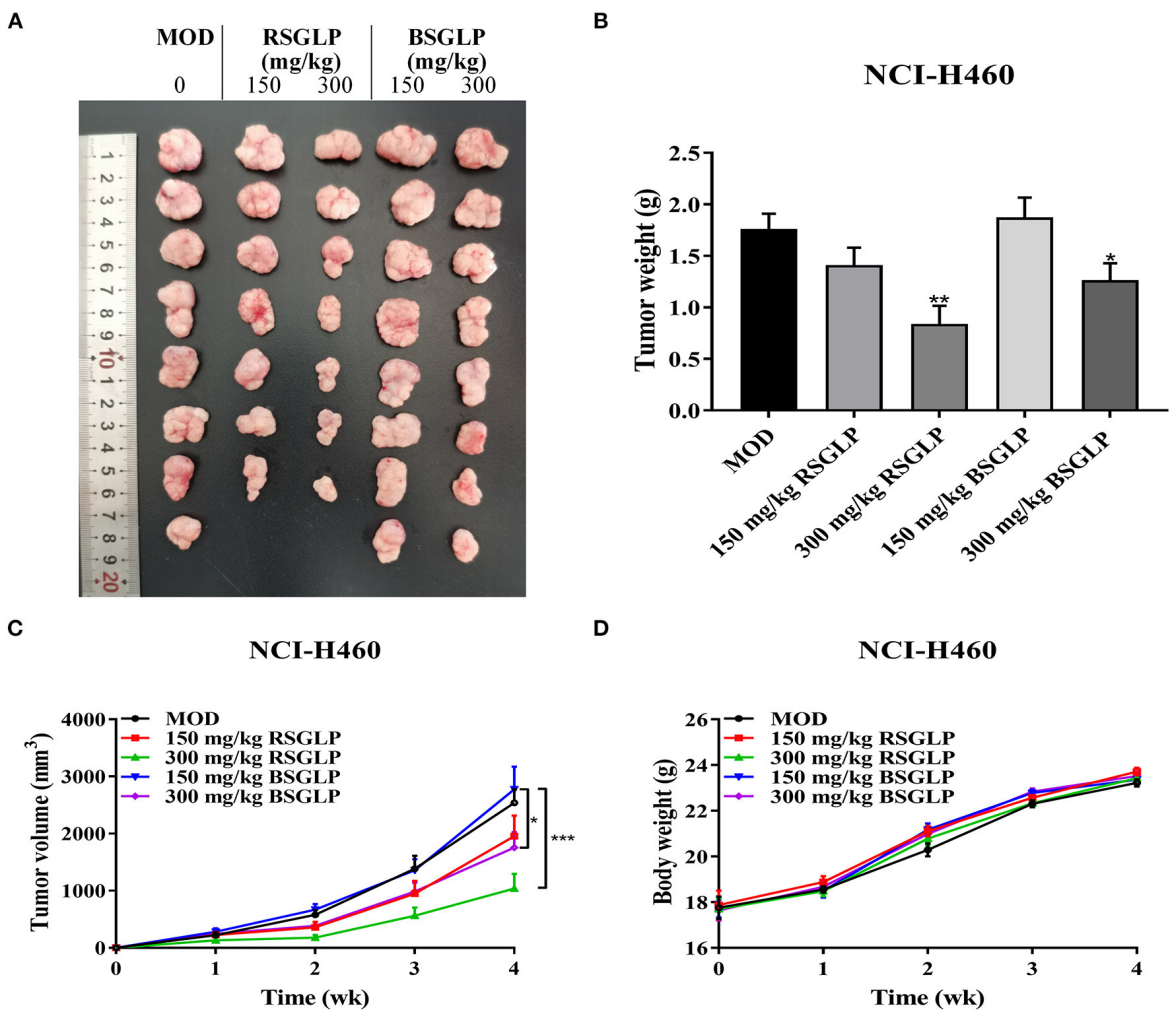
Comparison of the antitumor activity of RSGLP and BSGLP in NCI-H460 xenograft nude mice. **(A)** Image of the tumor samples from each group. **(B)** Final tumor weight of each group. **(C)** Growth curve of tumor volume of each group. **(D)** Body weight of each group. Nude mice were treated with saline (Con and MOD), RSGLP and BSGLP (150 and 300 mg/kg) for 4 wk. MOD: model. All values are presented as the mean ± SE (*n* = 7–8). *p < 0.05, **p < 0.01, compared with MOD group.

Next, we compared the antitumor effects of RSGLP and BSGLP using the four most GLP-sensitive cell lines in xenograft mouse model. However, the HepG2 cell line was described as non-tumorigenic at the ATCC's official website. Therefore, we did not conduct the HepG2 xenograft study, but only examined the antitumor effects of the two GLPs in HCT116, MDA-MB-231, and NCI-H460 xenograft mouse models. Unfortunately, tumor formation in MDA-MB-231 xenograft model was not successful after several pilot studies. We only observed very small tumor formation after injection of MDA-MB-231 cells (data not shown). On the contrary, both NCI-H460 and HCT116 cells were suitable for tumor formation in nude mice. As shown in Figures 4A–C, RSGLP significantly inhibited tumorigenesis in NCI-H460 xenograft model. Both low and high doses (150 and 300 mg/kg) of RSGLP suppressed the rate of tumor formation, and 7 out of 8 mice developed tumors. However, compared to the model group, the low dose BSGLP had no inhibitory effect on tumorigenesis in nude mice. High dose BSGLP significantly reduced tumorigenesis in NCI-H460 xenograft mice but was less potent than the high dose RSGLP in terms of tumor volume and final tumor weight (Figures 4A–C). The body weights of the mice were not affected by RSGLP or BSGLP treatment in the NCI-H460 xenograft mice (Figure 4D).

**Figure 5:**
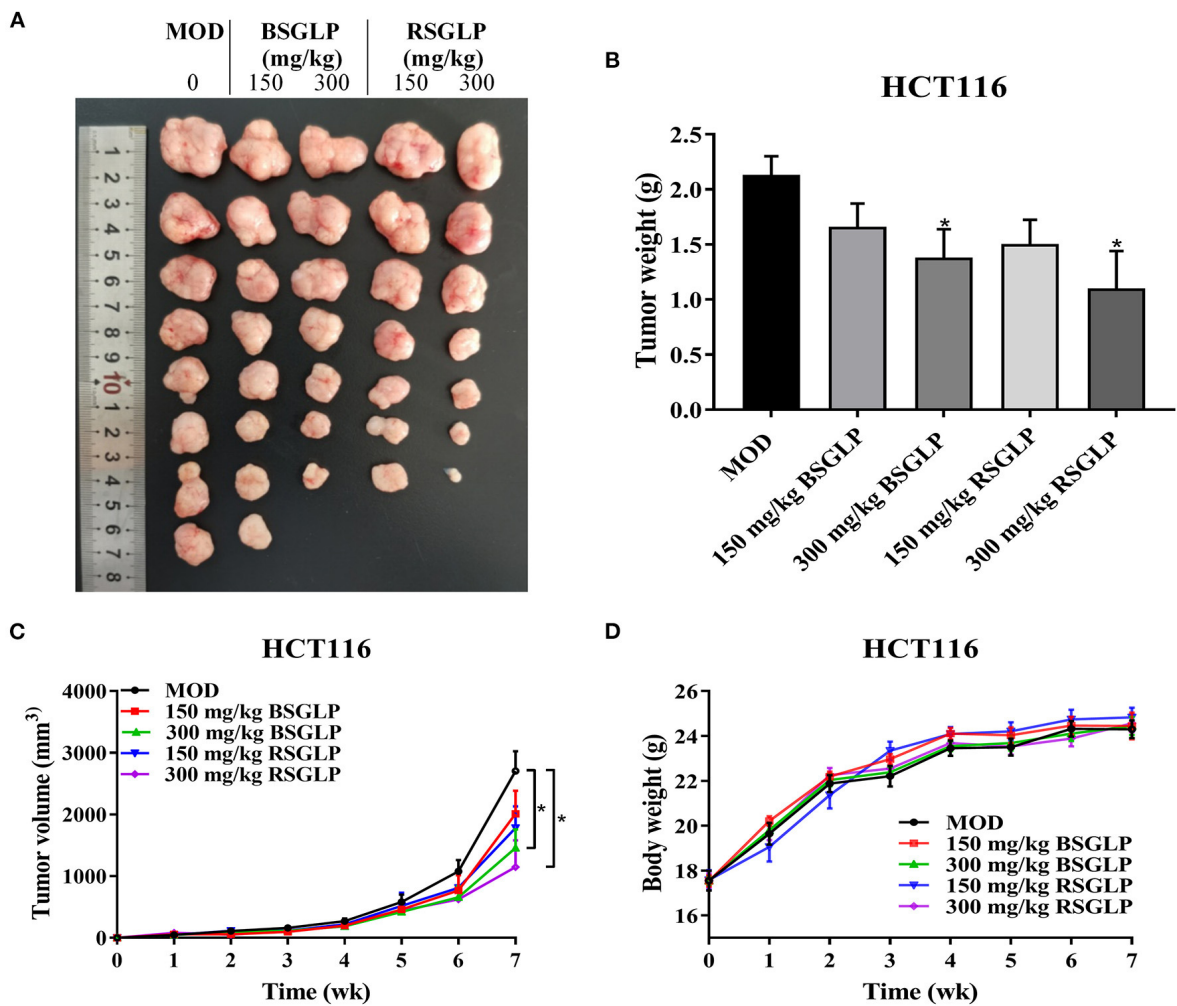
Comparison of the antitumor activity of RSGLP and BSGLP in HCT116 xenograft nude mice. **(A)** Image of the tumor samples from each group. **(B)** Final tumor weight of each group. **(C)** Growth curve of tumor volume of each group. **(D)** Body weight of each group. HCT116 xenograft nude mice were treated with saline (Con and MOD), BSGLP and RSGLP (150 and 300 mg/kg) for 7 wk. All values are presented as the mean ± SE (*n* = 7–8). *p < 0.05, compared with MOD group.

Similar to the NCI-H460 xenograft model, RSGLP and BSGLP inhibited tumorigenesis of the colon HCT116 xenografts in nude mice, and the inhibitory effect was dose-dependent (Figures 5A–C). Except for the low dose BSGLP, all other treatments suppressed the rate of tumor formation, and 7 out of 8 of mice developed tumors in each group. Both the high dose RSGLP and BSGLP significantly reduced tumorigenesis in terms of tumor volume and final tumor weight (Figures 5A–C). Similar to the NCI-H460 xenograft mice, the body weights of the nude mice were not affected by RSGLP or BSGLP treatment in HCT116 xenograft model (Figure 5D). Taken together, these results suggest that in line with the *in vitro* study, RSGLP is more potent than BSGLP in inhibiting xenograft tumor growth in nude mice.

### Comparison of the antiproliferation and pro-apoptotic effects of RSGLP and BSGLP *in vivo*

**Figure 6:**
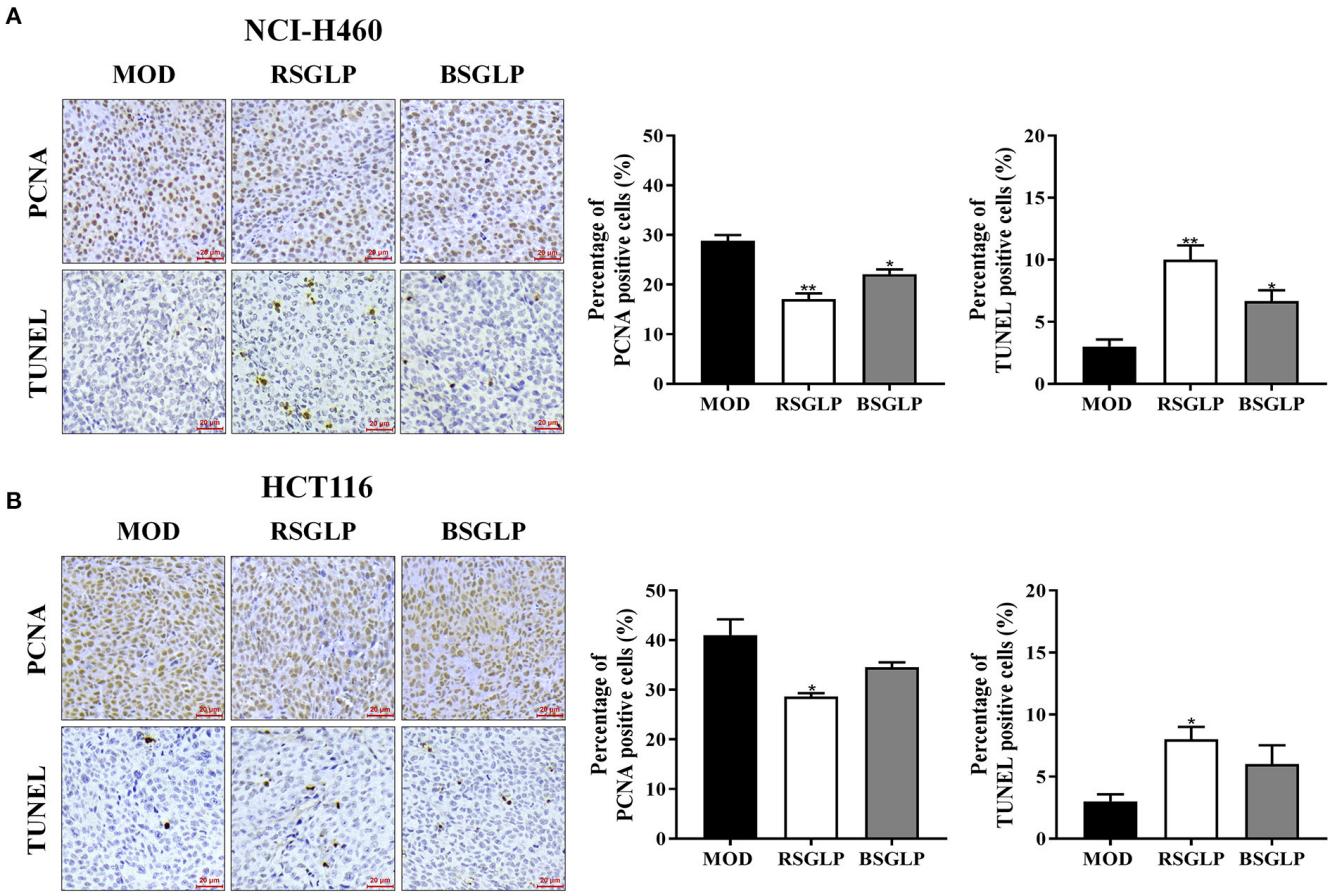
Comparison of the antiproliferative and pro-apoptotic effect of RSGLP and BSGLP in xenograft tumors as determined using IHC staining. PCNA and TUNEL staining in NCI-H460 **(A)** and HCT116 **(B)** xenograft tumor sections. Images were taken under an upright Metallurgical microscope. Scale bar: 20 μm. The percentage of positive cells was calculated by counting positive cells in tumor sections and then divided by total cells (300 cells per field, 3 fields of each section). All values are presented as the mean ± SE from 3 fields of each sample of a total of 3 samples per group. *p < 0.05, **p < 0.01, compared with MOD group.

Next, IHC was used to examine the effects of high dose RSGLP and BSGLP on the expression of PCNA, a cell proliferation marker. TUNEL, an approach to detect apoptosis, was used to compare the induction of apoptosis by the two GLPs in tumors. As shown in Figures 6A,B, compared with the model group, high dose RSGLP significantly reduced the expression of PCNA and increased the percentage of TUNEL positive cells in both NCI-H460 and HCT116 xenograft tumors, indicating that RSGLP inhibited cell proliferation and promoted apoptosis in both xenograft tumors. High dose BSGLP significantly reduced the expression of PCNA and increased the percentage of TUNEL positive cells only in NCI-H460 xenograft tumors, but the effect was less potent than that of RSGLP (Figures 6A,B). These results are consistent with the results from the *in vitro* experiment that RSGLP is more potent in inducing apoptosis and cell death than BSGLP.

### Comparison of the inhibitory effects of RSGLP and BSGLP on splenomegaly and inflammation

**Figure 7:**
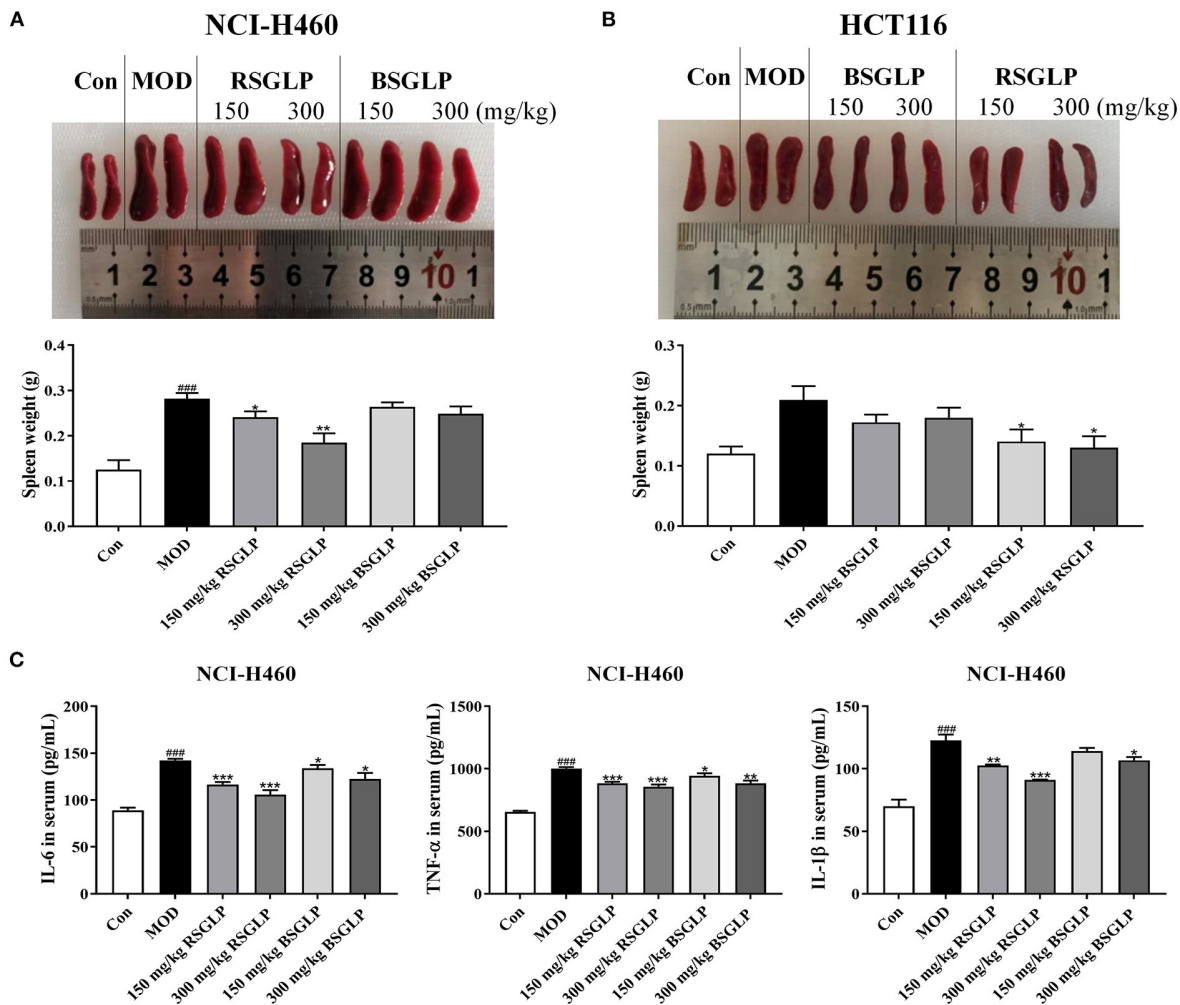
Comparison of the inhibitory effects of RSGLP and BSGLP on splenomegaly and inflammation in xenograft nude mice. Image of the spleen samples and spleen weight of the NCI-H460 **(A)** and HCT116 **(B)** xenograft nude mice. **(C)** Serum levels of inflammatory cytokines IL-6, IL-1β, and TNF-α in the NCI-H460 xenograft nude mice. All values are presented as the mean ± SE (*n* = 7–8). *p < 0.05, **p < 0.01, ***p < 0.001, compared with MOD group. ^###^*p* < 0.001, compared with Con group.

A number of earlier studies have reported that spleen enlargement, or splenomegaly, is an inevitable consequence of tumor growth in tumor-bearing nude mice regardless of drug treatments (30, 31). We observed that the weights of spleen were significantly increased in NCI-H460 xenograft mice at the end of the study (Figure 7A). However, RSGLP at both concentrations, significantly inhibited splenomegaly in NCI-H460 xenograft model. Different from the NCI-H460 xenograft model, splenomegaly in HCT116 xenograft was less obvious but still significantly enlarged (Figure 7B). Both doses of RSGLP also significantly inhibited splenomegaly in HCT116 xenograft nude mice (Figure 7B). However, in both xenograft models, BSGLP slightly decreased splenomegaly, but not at significant level (Figures 7A,B).

Spleen, as the largest secondary lymphoid organ in body, exerts a significant effect in regulating the inflammation-related immune responses (32). It has been reported that the triggering of excessive inflammation leads to the pathogenesis and progression of splenomegaly in the mice (29). Therefore, we examined the serum levels of the pro-inflammatory cytokines IL-1β, IL-6, and TNF-α in NCI-H460 xenograft model, which has the best response to GLPs in terms of tumor and splenomegaly inhibition. As shown in Figure 7C, compared with the control, the serum level of these cytokines in the model group was significantly upregulated. However, both RSGLP and BSGLP treatment decreased the level of these cytokines (Figure 7C). In addition, the effect of RSGLP is more significant than that of BSGLP, suggesting RSGLP is more effective than BSGLP in inhibiting systematic inflammation in the nude mice.

### Comparison of the anti-inflammation effect of RSGLP and BSGLP in RAW264.7 cells

As an important part of the innate immune system, macrophages play a key regulatory role in immune defense, inflammation regulation, tissue repair, maintenance of metabolic balance, and carcinogenesis (33, 34). LPS-induced macrophage activation has been widely used to study the anti-inflammation and immunoregulatory effects of anticancer agents *in vitro*. We further studied and compared the inhibitory effect of the two GLPs on inflammation using LPS-stimulated macrophage RAW264.7 model. We first identified the concentrations of RSGLP and BSGLP in RAW264.7 cells that had no cytotoxicity effects using MTT assay. As shown in the Supplementary Figure 3, both GLPs within 0–2.5 mg/mL demonstrated no cytotoxic effects on RAW 264.7 cells. Next, the same concentrations of RSGLP and BSGLP (0–2.5 mg/mL) were used to compare the effects of the two GLPs on inflammation.

**Figure 8:**
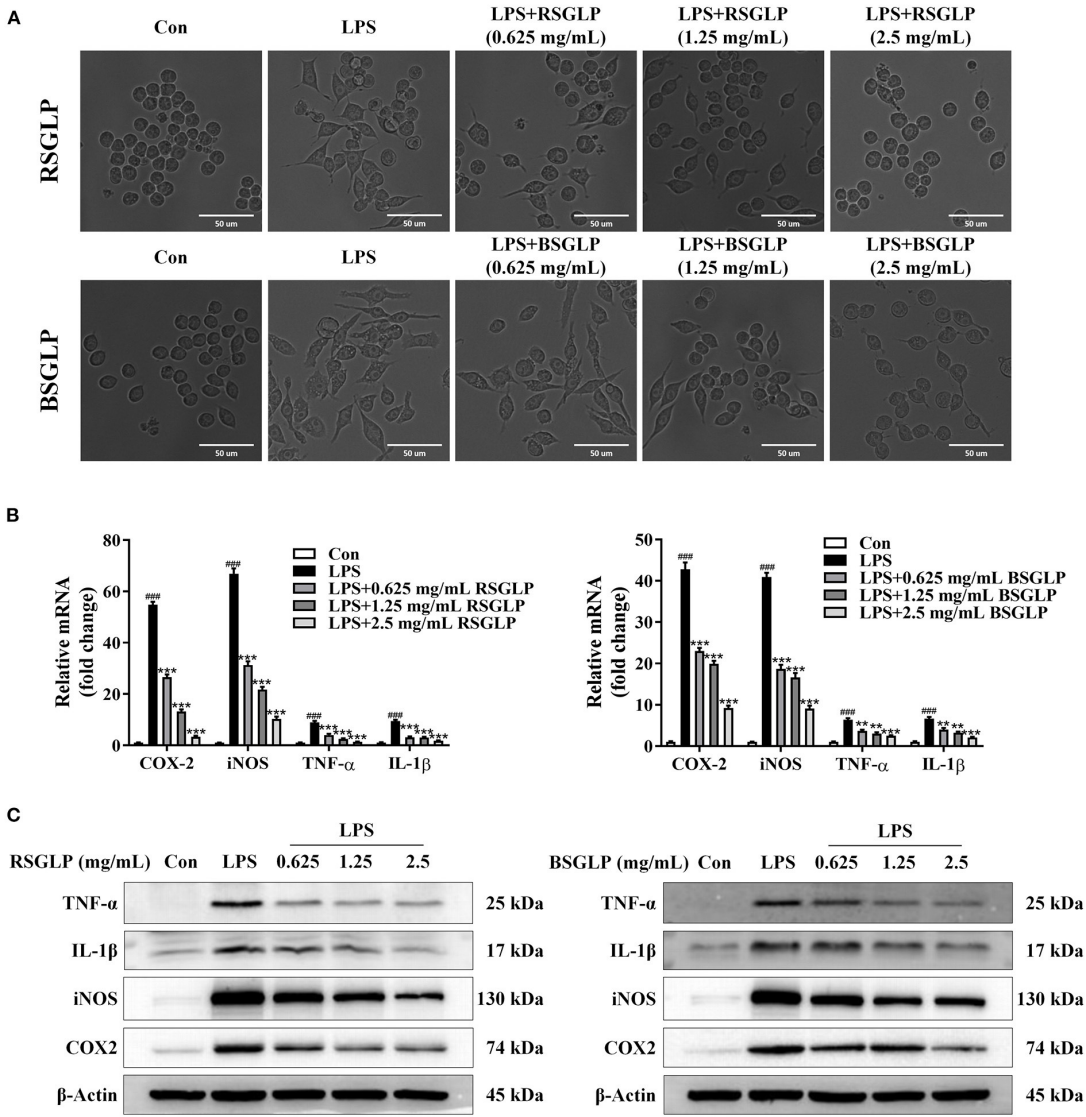
Comparison of the effects of RSGLP and BSGLP on LPS-induced macrophage activation and inflammation in RAW264.7 cells. **(A)** Morphological alteration of RAW264.7 cells. **(B)** The expressions of inflammation-related genes *iNOS, COX-2, TNF-*α, and *IL-1*β as determined using qRT-PCR. **(C)** The expressions of inflammation-related proteins iNOS, COX-2, TNF-α, and IL-1β as determined using Western blotting. Cells were treated with LPS (1 μg/mL) and GLP (RSGLP or BSGLP) at 0–2.5 mg/mL for 24 h. Data are presented as mean ± SE from three independent experiments. ***p < 0.001, compared with LPS treatment group. ^###^*p* < 0.001, compared with Con group.

**Figure 9:**
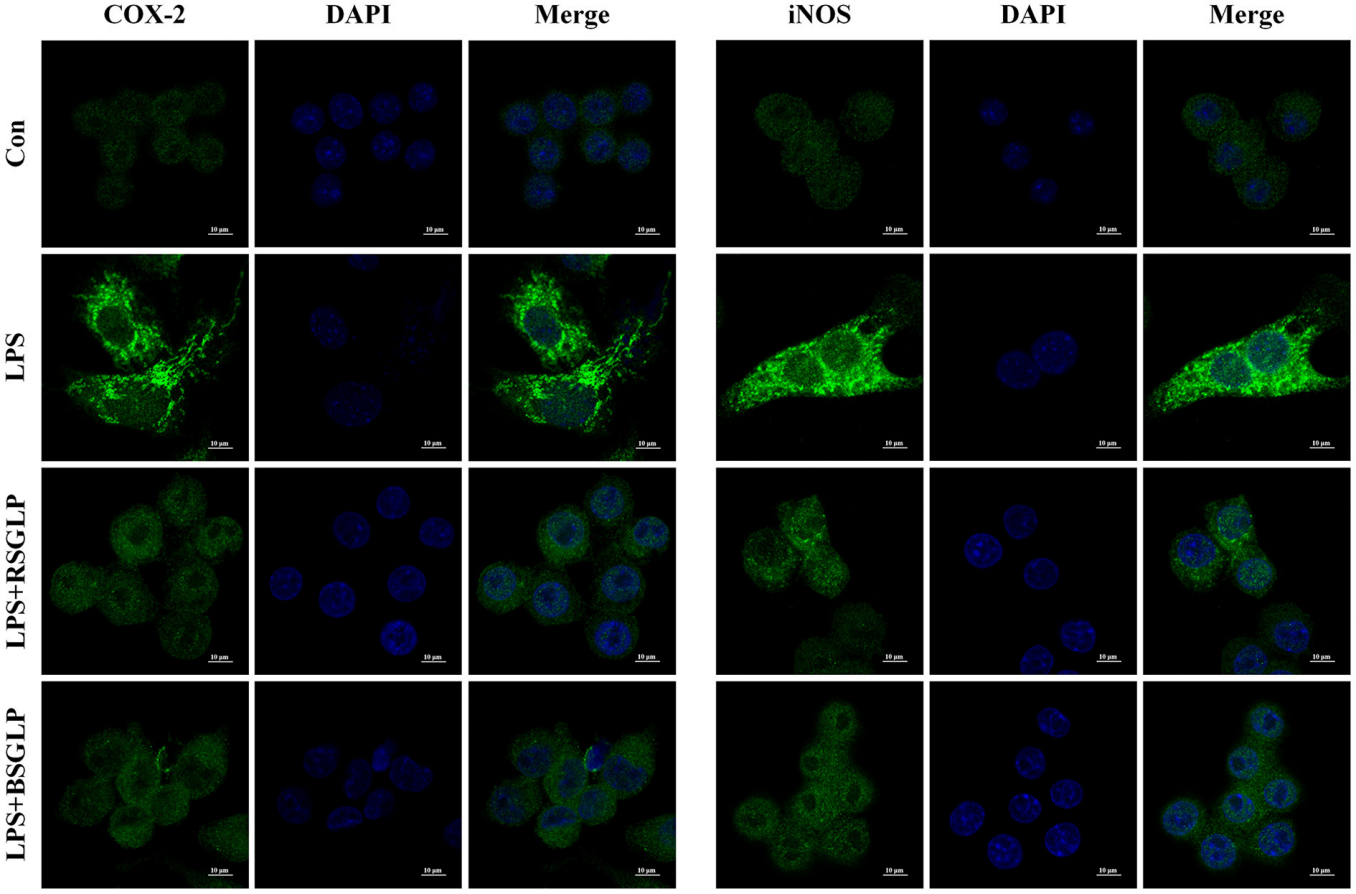
Immunofluorescence staining of iNOS and COX-2 in RAW264.7 cells cotreated with LPS (1 μg/mL) and RSGLP or BSGLP (both at 1.25 mg/mL).

As shown in Figure 8A, LPS activated macrophage RAW 264.7 cells as manifested by increase in cell size and distinct dendritic morphologic change. However, Both RSGLP and BSGLP reversed LPS-induced morphological change in RAW 264.7 cells in a dose-dependent manner (Figure 8A), suggesting that both GLPs inhibited macrophage activation. The anti-inflammatory activity of GLPs was then determined. Compared with the control cells, the expressions of the pro-inflammatory cytokines or mediators *IL-1*β, *TNF-*α, *iNOS*, and *COX-2* were significantly increased upon LPS treatment at the mRNA level (Figure 8B). However, both GLPs significantly reduced LPS-induced upregulation of these genes in a dose-dependent manner with RSGLP had more dramatic effects than the BSGLP (Figure 8B). Western blotting further confirmed that both GLPs inhibited LPS-induced overexpression of IL-1β, TNF-α, iNOS, and COX-2 at the protein level (Figure 8C). As shown in the Supplementary Figure 4, densitometry analysis revealed that RSGLP is more potent in inhibiting the expressions of LPS-induced overexpression of IL-1β, TNF-α, iNOS, and COX-2 proteins than BSGLP in RAW 264.7 cells. Immunofluorescence assay further confirmed that both GLPs (1.25 mg/mL) could inhibit the up-regulation of COX-2 and iNOS in RAW 264.7 cells that induced by LPS (Figure 9). Taken together, these results suggest that both RSGLP and BSGLP could regulate the immune response by inhibiting the activation of macrophage RAW 264.7 cells and the expressions of the inflammatory cytokines or mediators. In addition, these results also suggest that the anti-inflammatory effect of RSGLP was stronger than that of BSGLP when comparing at the same concentrations.

## Discussion

It has been well-reported that GLPs elicit the anti-cancer effects by various mechanisms, such as anti-proliferative, pro-apoptotic, anti-metastatic, anti-angiogenic, anti-inflammatory, and immunomodulatory activities (11, 12). RSGLP is a new generation of the *G. lucidum* product, which removed the sporoderm completely from the spores. In the current study, we first compared the extraction yields between RSGLP and BSGLP, then characterized the chemical properties of the two GLPs. Our results revealed that RSGL which removed the sporoderm completely yielded much more polysaccharide than BSGL. It has been reported that the sporoderm of *G. lucidum* spores is composed mainly of chitin, calcium, and silicon (22). Removing sporoderm from spores leads to the high yield of extracted polysaccharides and this result indicates that the removal of sporoderm is beneficial to yield polysaccharides from *G. lucidum* spores. Similarly, RSGL has been reported that contains a higher amount of triterpenoids and polysaccharides than BSGL (27, 35). We further characterized the chemical properties of the two GLPs. The chemical property of RSGLP has never been reported. Our results revealed that both GLPs are heteropolysaccharides composed of arabinose, mannose, glucose, and galactose with an average molecular weight of 66.8 and 33.1 kDa, respectively. Both GLPs contains the highest proportion of glucose, while RSGLP has more glucose, but less mannose and arabinose than BSGLP.

At present, a number of studies have reported the anticancer effects of GLP in many cancer cells. However, for most of the study, only one cancer type was examined, and no study has screened the anticancer effects of GLP in multiple cancer types using multiple cell lines. We compared the anticancer effects of RSGLP and BSGLP in eight cancer cell lines that represent colon, liver, lung, and breast cancers. Our results demonstrate that RSGLP is more potent than BSGLP in inhibiting cancer cell viability and in inducing apoptosis in the eight cancer cell lines. We found that within the same cancer type, different cell line responded differently to GLP treatment. The colon HCT116, liver HepG2, lung NCI-H460 and breast MDA-MB-231 cancer cells are more sensitive to GLPs than colon HT-29, liver HuH-7, lung A549, and breast MCF-7 cancer cells line. Our results also demonstrate that RSGLP is more potent than BSGLP in inhibiting tumor growth, splenomegaly, and inflammation. We further found that RSGLP demonstrates more potent anti-inflammation activity than BSGLP in LPS-treated macrophage RAW 264.7 cells. Similarly, a recent study reported that the immunomodulatory function of triterpenoids extracted from RSGL was higher than that of BSGL (27). To our knowledge, this is the first study that compared the chemical properties between RSGLP and BSGLP and examined the anti-cancer effects and potential molecular mechanisms of RSGLP and BSGLP in a broad range of cancer types. Our results revealed that the newly developed technology that producing the sporoderm-removed spores of *G. lucidum* may serve as a promising new generation of *G. lucidum* products.

It is well-acknowledged that the biological activities of polysaccharides are affected by Mw, monosaccharide composition, glycosidic linkage patterns, configuration (α or β), degree of branching, and length of branch, etc. (36–38). In this study, we examined the Mw and monosaccharide composition of the two GLPs, which we think are the two most important factors that differing in RSGLP and BSGLP. Similarly, the Mw and monosaccharide composition were considered to be the two major factors affecting the therapeutic action of polysaccharides extracted from *Cordyceps sinensis* (39). Besides, many studies also suggested that different Mw could affect the biological activity of the polysaccharides (11, 36, 38, 40–42).

The ineffective wall components were removed in RSGL, thus RSGL contains a higher amount of polysaccharides compared to BSGL. The HPGPC results showed that the Mw of RSGLP is higher than BSGLP, which is probably due to the sporoderm removal technique promoted the release of polysaccharides with a high Mw in spores. It is well-known that the cell walls of the plants contain a large amount of polysaccharides (43). By removing these polysaccharides from the cell wall of the spores, the composition of monomers was also changed. Meanwhile, the Mw of a polymer is profoundly affected by the degree of polymerization (44). The higher Mw of the polysaccharide in RSGL than in BSGL may also be due to the different degrees of polymerization of the monomers, which needs to be further examined in future studies. As mentioned above, it has been reported that within a certain range of Mw, higher Mw of polysaccharides extracted from *G. lucidum* demonstrates stronger immunomodulatory and antitumor activity than polysaccharides with lower Mw (36, 38, 40–42). For examples, it was reported that higher Mw of GLP (Mw >8–10 kDa) showed stronger antioxidant and antiproliferative activities than GLP with lower Mw (<8 kDa) in Rat PC12 pheochromocytoma cells (40). An earlier study also found that GLP with higher Mw was more effective in inhibiting sarcoma 180 xenograft tumor growth in ICR/Slc mice (41).

In our study, we found that RSGLP has an average Mw of 66.8 kDa, which is almost doubled than that of BSGLP, which is 33.1 kDa. However, different from our study, the Mw of polysaccharide in BSGL obtained from Green Valley Company (Shanghai, China) was reported to be 8 kDa (45). The Mw of polysaccharide in the BSGL from Guangdong Yuewei Edible Fungi Technology company (Guangdong, China) was reported to be 3.6 kDa (46). Our recent study suggested that BSGL obtained from another resource (Shandong Zhengxin bio, Shandong, China) has an average molecular weight of 26.0 kDa (47). While GLP extracted from BSGL that provided by Jiangsu Alphay Biological Technology Co., Ltd. (Nantong, China) has an average Mw of 108 kDa (48). These results suggest that Mw of BSGL can be affected by source of the mushroom. Similarly, we expect that the chemical properties, such as Mw, and the biological properties of RSGLP could also be affected by the source of the mushrooms.

In addition, it is well-known that natural polymers such as polysaccharides possess inherent biocompatibility and biodegradability, and thus have been widely used for drug delivery system, including anti-cancer drugs (49, 50). It was reported that even subtle differences in polymer Mw (such as a few kilodaltons) can affect the efficiency of the drug delivery system using polysaccharides (51). At present, there is no consensus on exactly what Mw of the polymers is optimal for certain drug delivery. Therefore, more studies are needed to compare the efficiency when applying purified BSGLP or RSGLP as material for drug delivery system.

Our *in vivo* study found that both GLPs, in particular RSGLP, showed potent tumor inhibitory effects in the xenograft nude mice. An interesting observation we found was that the tumor growth rate of the NCI-H460 xenograft was faster than that of the HCT116 xenograft. Within 4 week, NCI-H460 xenograft tumors can reach 2.0 cm in length or width in the control group. While it took about 7 wk for HCT116 xenograft to reach this size. In addition, during the experiment, there were no significant differences in tumor growth among all groups in the first 2 weeks in the NCI-H460 xenografts, but from the third week, compared with the model, RSGLP and high dose BSGLP treatment significantly inhibited the tumor growth rate. In the HCT116 xenografts, there was no significant difference in tumor volume between the treatment and the model groups in the first 5 weeks. From the 6th week, the growth rate of tumor volume in the high dose GLPs feeding groups was significantly lower than that of the model group. These results indicate that better effects in inhibiting tumorigenesis by GLP may be achieved through prolonged treatment. However, due to the largest tumor in each group has reached 2 cm, we had to euthanize the mice at 4 wk for NCI-H460 or 7 wk for HCT116 xenografts. Our previous study also suggests that GLP extracted from a different source of *G. lucidum* may be more effective in inhibiting colorectal tumor progression at late-stage but not being quite effective in inhibiting tumor development at early-stage in HCT116 xenograft model (25). Previous clinical studies found that *G. lucidum* treated for long-term is more effective than short-term treatment to reduce the death rate in late-phase cancer patients (52). Available clinical studies in general suggest that *G. lucidum* is safe to either normal population or patients with different health conditions (53, 54). However, at present, the safety and potential toxicity of long-term use of RSGLP for therapeutic purpose or prevention purposes have not to be examined, which needs to be further evaluated in future studies.

Splenomegaly has been reported in early studies to be phenomenal in nude mice bearing xenograft tumors and which is closely associated with tumor growth and progression (55–58). It was reported that in nude mice that bearing tumors, with tumor growth, spleen enlargement was accompanied by accumulation of macrophages and neutrophils simultaneously in the spleen, with a decrease in T cells and most cases also in B cells (31). Another study also reported that nude mice bearing tumors showed marked splenomegaly and neutrophilia (30). They found that splenomegaly was accompanied by a marked expansion of hematopoietic spaces and atrophy of lymphoid follicles (30). Other than reported in tumor-bearing nude mice, splenomegaly has also been reported in C57BL/6 mice that developed lymphoma especially at later or more advanced stages (59). Le et al. reported that early gemcitabine treatment significantly inhibited tumor growth, reduced splenomegaly, and significantly decreased the proportion of the myeloid-derived suppressor cells (MDSC) in the spleen of the 4T1 tumor-bearing nude mice (60). As the spleen is an important immune organ outside of the tumor microenvironment, the role of the spleen in tumor-host interaction and tumor progression upon RSGLP treatment needs to be further studied in the future.

Tumorigenesis is a complex process, the immune system, as the body's defense system, can effectively recognize foreign substances and remove them through adaptive or acquired immune responses (61). Chronic inflammation and the level of inflammatory mediators play an important role in the initiation, development, and progression of cancer (2). As an important part of the innate immune system, macrophages play a key regulatory role in immune defense, inflammation regulation, tissue repair, maintenance of metabolic balance, and carcinogenesis (33, 34). When inflammation occurs, pro-inflammatory macrophages further promote their accumulation in the damaged tissue through self-proliferation, and then secrete a variety of inflammatory factors, such as TNF-α and IL-1β to create an inflammatory environment and promote carcinogenesis (62). In this study, we found that both RSGLP and BSGLP treatment reduced the expression of inflammatory cytokines IL-6, IL-1β, and TNF-α in serum of the xenograft mice. In addition, both GLPs inhibited LPS-induced RAW264.7 cells activation and overexpression of IL-6, IL-1β, iNOS, and COX-2. Regardless *in vivo* or *in vitro*, RSGLP demonstrated more potent anti-inflammatory effects than BSGLP. Many studies suggest that polysaccharide was the most important component responsible for the immunoregulatory effects of *G. lucidum* against many diseases (17, 63–65). Studies have demonstrated that GLP can improve the body's immunity (17, 66), and which may be responsible for its anti-cancer effects (67–69). Other than the anti-inflammatory effects of RSGLP in macrophage cells, the immunoregulatory role of RSGLP in regulating other immune cells, such as T cells, B lymphocytes, and natural killer cells, needs to be further examined in future studies.

Previously, we reported that BSGLP induced apoptosis in HCT116 cells by regulating the expressions of apoptosis-related protein Bcl-2, PARP, caspase-3, and BAX (25). More recently, we reported that BSGLP promoted apoptosis by inducing autophagosome accumulation and inhibiting autophagy flux, which was mediated by MAPK/ERK signaling pathway (26). Another study from our laboratory showed that BSGLP can induce apoptosis in human prostate cancer cell line PC-3 cells by mediating the activation of non-steroidal anti-inflammatory drug activation gene 1 (NAG-1) (18). However, the exact molecular targets and signaling pathways that regulate RSGLP-induced apoptosis need to be further studied in the future.

## Conclusion

In conclusion, this study demonstrated that the RSGL, which removed the sporoderm completely form BSGL yielded much more polysaccharide than the BSGL. The Mw of RSGLP is higher than the Mw of BSGLP. Although both GLPs are heteropolysaccharides that composed of arabinose, mannose, glucose, and galactose, RSGLP contained a much higher ratio of glucose than BSGLP. We further demonstrated that RSGLP has much higher dose-efficacy than BSGLP in inhibiting cell viability or inducing apoptosis in eight cancer cell lines representing colon, liver, breast, and lung cancers *in vitro*. The sensitivity of cancer cells to both GLP treatments differed within same cancer type and differed among different cancers. *In vivo* studies revealed that RSGLP is more effective in inhibiting NCI-H460 and HCT116 xenograft tumor growth. The higher content of polysaccharides and different chemical properties in the RSGLP could be the reason for its better effect than BSGLP for the anticancer effects. RSGLP is also more effective in inhibiting tumor-induced spleen enlargement in NCI-H460 and HCT116 xenograft models, suggesting an improvement of immunity by RSGLP in nude mice. We further demonstrate that RSGLP is more potent than BSGLP in inhibiting LPS-induced overexpression of inflammatory mediators in RAW264.7 cells. Collectively, our study suggests that RSGL and RSGLP could serve as promising anticancer agents, which represent a new generation of the *G. lucidum* product, for research in food nutrition, agricultural, and pharmaceutical sciences.

## Data availability statement

The raw data supporting the conclusions of this article will be made available by the authors, without undue reservation.

## Ethics statement

The animal study was reviewed and approved by Committee on the Ethics of Animal Experiments of Zhejiang Chinese Medical University.

## Author contributions

LF: data curation, investigation, software, validation, visualization, and writing—original draft. QZ: data curation, investigation, and software. CuG, DG, ChG, CC, RC, YW, and JC: investigation and data curation. ZL: data curation and software. JX: data curation and software. TS: data curation and visualization. JW: conceptualization, data curation, visualization, software, and methodology. XW: conceptualization, data curation, software, writing—review and editing, investigation, visualization, formal analysis, methodology, project administration, resources, supervision, and funding acquisition. All authors contributed to the article and approved the submitted version.

## Funding

This study was funded by the Key Foundation of Science and Technology Department of Zhejiang (2019C02100) and the National Natural Science Foundation of China (81973521).

## Conflict of interest

The authors declare that the research was conducted in the absence of any commercial or financial relationships that could be construed as a potential conflict of interest.

## Publisher's note

All claims expressed in this article are solely those of the authors and do not necessarily represent those of their affiliated organizations, or those of the publisher, the editors and the reviewers. Any product that may be evaluated in this article, or claim that may be made by its manufacturer, is not guaranteed or endorsed by the publisher
