# Supplementary material for: Removing the sporoderm from the sporoderm-broken spores of Ganoderma lucidum improves the anticancer and immune-regulatory activity of the water-soluble polysaccharide
Source: Front Nutr. 2022 Sep 16;9:1006127. doi: 10.3389/fnut.2022.1006127 (PMC9524850; doi:10.3389/fnut.2022.1006127)
Supplement: Supplementary file 1 [file Data_Sheet_1.docx]

Supplementary Material

**Table S1.**

Primers used for qRT-PCR

| Protein | Gene | Primer Sequence |
| --- | --- | --- |
| TNF-α | *Tnfα* | Forward: 5'- ACGGCATGGATCTCAAAGAC-3'  Reverse: 5'- AGATAGCAAATCGGCTGACG-3' |
| IL-1β | *Il-1β* | Forward: 5'- TTGAAGAAGAGCCCATCCTC-3'  Reverse: 5'- CAGCTCATATGGGTCCGAC-3' |
| COX-2 | *Ptgs2* | Forward: 5'- TGAGCAACTATTCCAAACCAGC -3'  Reverse: 5'- GCACGTAGTCTTCGATCACTATC -3' |
| iNOS | *Nos2* | Forward: 5’- GGAGTGACGGCAAACATGACT -3′  Reverse: 5’- TCGATGCACAACTGGGTGAAC -3′ |
| β-actin | *β-actin* | Forward: 5'- CAGCTTCTTTGCAGCTCCTT -3'  Reverse: 5'- CACGATGGAGGGGAATAAG -3' |

**A**

**D**

**C**

**B**

**Figure S1**. Comparison of the cytotoxic effects of RSGLP and BSGLP in colon, liver, breast, and lung cancer cells as determined using MTT assay. **(A)** HT-29. **(B)** HuH-7. **(C)** MCF-7. **(D)** A549. Cells were treated with different concentrations of RSGLP (0, 1.25, 1.875, 2.5, and 3.125 mg/mL) and BSGLP (0, 5, 7.5, 10, and 12.5 mg/mL) for 24, 48, and 72 h. Data are presented as mean ± SE from three independent experiments. ^*^*p* < 0.05, ^**^*p* < 0.01, ^***^*p* < 0.001, compared with control group.

**Table S2**

|  | | IC_50_ (mg/mL) | | | |
| --- | --- | --- | --- | --- | --- |
|  |  | HT-29 | HuH-7 | MCF-7 | A549 |
|  | 24 h | － | － | － | － |
| RSGLP | 48 h | 2.819 ± 0.79 | 3.211 ± 1.03 | － | 15.48 ± 1.78 |
|  | 72 h | 2.028 ± 0.65 | 3.043 ± 1.22 | － | 6.667 ± 1.54 |
|  | 24 h | － | － | － | － |
| BSGLP | 48 h | 24.18 ± 2.35 | － | － | － |
|  | 72 h | 18.34 ± 1.87 | 16.84 ± 2.12 | 13.13 ± 1.98 | 9.213 ± 1.76 |
|  | 24 h | － | － | － | － |
| fold^a^ | 48 h | 8.58 | － | － | － |
|  | 72 h | 9.04 | 5.53 | － | 1.36 |

Half-maximal inhibitory concentration (IC_50_) of RSGLP and BSGLP against the human cancer cell lines (HT-29, HuH-7, MCF-7, and A549).

All experiments were performed in triplicates and reported as mean ± SE.

“-” The IC_50_ could not be calculated under the current treatment condition.

“^a^” The IC_50_ of BSLGP divided by the IC_50_ of RSGLP at the same time point for each cell line.

**Figure S2**. Comparison of the pro-apoptotic effects of RSGLP and BSGLP in colon, liver, breast, and lung cancer cells as determined using flow cytometry. **(A)** HT-29. **(B)** HuH-7. **(C)** MCF-7. **(D)** A549. Cells were treated with different concentrations of RSGLP (0, 1.875, 2.5, and 3.125 mg/mL) and BSGLP (0, 7.5, 10, and 12.5 mg/mL) for 48 h. The bottom panel is the total percentages of cell apoptosis. Data are presented as mean ± SE from three independent experiments. ^*^*p* < 0.05, ^**^*p* < 0.01, compared with control group.

**D**

**C**

**B**

**A**

**BSGLP**

**RSGLP**

**PI**

**PI**

**BSGLP**

**RSGLP**

**BSGLP**

**RSGLP**

**BSGLP**

**RSGLP**

**PI**

**PI**

**Con**

**7.5 mg/mL**

**10 mg/mL**

**12.5 mg/mL**

**Con**

**1.875 mg/mL**

**2.5 mg/mL**

**3.125 mg/mL**

**Con**

**7.5 mg/mL**

**10 mg/mL**

**12.5 mg/mL**

**Con**

**7.5 mg/mL**

**10 mg/mL**

**12.5 mg/mL**

**Con**

**1.875 mg/mL**

**2.5 mg/mL**

**3.125 mg/mL**

**Con**

**1.875 mg/mL**

**2.5 mg/mL**

**3.125 mg/mL**

**Con**

**7.5 mg/mL**

**10 mg/mL**

**12.5 mg/mL**

**Con**

**1.875 mg/mL**

**2.5 mg/mL**

**3.125 mg/mL**

**Annexin V**

**HT-29**

**Annexin V**

**HuH-7**

**Annexin V**

**MCF-7**

**Annexin V**

**A549**

**Figure S3**. Comparison of the cytotoxic effects of RSGLP and BSGLP on RAW264.7 cells as determined using MTT assay. Cells were treated with different concentrations of RSGLP and BSGLP (0, 0.625, 1.25, and 2.5 mg/mL) for 24h. Data are presented as mean ± SE from three independent experiments.

**Figure S4**. The relative intensities of inflammation-related proteins (iNOS, COX-2, TNF-α, and IL-1β) after normalized against β-Actin. Cells were treated with LPS (1 μg/mL) and GLP (RSGLP or BSGLP) at 0-2.5 mg/mL) for 24 h. Data are presented as mean ± SE from three independent experiments. ^*^*p* < 0.05, ^**^*p* < 0.01, ^***^*p* < 0.001, compared with LPS treatment group. ^###^*p* < 0.001, compared with Con group.
